# Exploring Sulpiride as an Alternative to Testosterone Propionate for Inducing Benign Prostatic Hyperplasia in Rodent Models

**DOI:** 10.3390/toxics14020180

**Published:** 2026-02-18

**Authors:** Solomon Owumi, Esther M. Pius, Hikmah A. Abdulganiyu, Ifeoluwa O. Alabi, Victor O. Eso, Abdullah A. Sanusi, Oluwaseun M. Owolabi, Uche O. Arunsi, Jesutosin O. Babalola, Moses T. Otunla, Ayomide P. Akomolafe, Emiola O. Olapade-Olaopa, Adegboyega K. Oyelere, Olorunseun O. Ogunwobi, Chima M. Amadi

**Affiliations:** 1 Cancer Research and Molecular Biology Research Laboratories, Department of Biochemistry, University of Ibadan, Ibadan 200005, Oyo State, Nigeria; piusmayowaesther@gmail.com (E.M.P.); ganiyuadeola540@gmail.com (H.A.A.); alabiifeoluwa10@gmail.com (I.O.A.); thevictoreso@gmail.com (V.O.E.); sanuxabd@gmail.com (A.A.S.); oluwaseunmartinsowolabi@gmail.com (O.M.O.); 2 School of Chemistry and Biochemistry, Georgia Institute of Technology, Atlanta, GA 30332, USA; venniabia@gmail.com; 3 Nutrition and Industrial Biochemistry Research Laboratories, Department of Biochemistry, University of Ibadan, Ibadan 200005, Oyo State, Nigeria; jesutosinbabalola@gmail.com; 4 Department of Pharmacology and Nutritional Sciences, College of Medicine, University of Kentucky, Lexington, KY 40536, USA; 5 Department of Biochemistry and Molecular Biology, University of Nebraska Medical Centre, Omaha, NE 68198, USA; peterayomide13@gmail.com; 6 Department of Surgery, College of Medicine, University of Ibadan, Ibadan 200004, Oyo State, Nigeria; okeoffa@gmail.com; 7 Department of Chemistry and Biochemistry, Michigan State University, Lansing, MI 48824, USA; olorunseun@yahoo.com; 8 Dedra Nutraceuticals and Biotechnology Initiative, Garki 900024, Abuja FCT, Nigeria; matthewamadic@outlook.com

**Keywords:** benign prostatic hyperplasia, testosterone propionate, sulpiride, hyperprolactinemia, androgen receptor, rodent models

## Abstract

Benign prostatic hyperplasia (BPH) is a significant health issue among ageing men, with ongoing research focused on elucidating its underlying mechanisms and improving experimental models. Testosterone Propionate (TP) is the first line of choice for the induction of BPH in experimental rodent models. However, TP’s controlled status as a Schedule III drug in the United States and a Class C drug in the UK presents challenges in obtaining TP for experimental use, giving preference to the sulpiride model since it is easily obtained as an alternative for the induction and study of BPH. A comprehensive literature search was conducted across multiple electronic databases, including PubMed/MEDLINE, Embase, and Web of Science. The primary PubMed search strategy included combinations of Medical Subject Headings (MeSH) and free-text terms: (“Benign prostatic hyperplasia induction” OR “and rodent models’’) AND (“Testosterone Propionate model”) AND (“sulpiride model”). Studies were included if they induced BPH (using testosterone or sulpiride models). Titles and abstracts were screened for relevance; eligible articles underwent full-text review, with data extracted thematically. No formal risk-of-bias scoring was used due to the narrative approach; instead, studies were appraised by design, rigor, plausibility, and evidence. This study reviewed published and publicly available data, so no ethical approval was required. Although both TP and sulpiride induce BPH via various mechanisms, this review provides a comparative analysis of these two commonly utilised models for studying BPH. In the TP approach, castrated rodents receive daily subcutaneous injections for 4 weeks, resulting in dihydrotestosterone (DHT)-mediated epithelial hyperplasia predominantly affecting the ventral prostate lobes. Conversely, the sulpiride model is non-invasive, employs intact animals treated with sulpiride, and induces hyperprolactinemia-mediated BPH via interactions with androgen and oestrogen receptor pathways that stimulate prostatic stromal and epithelial proliferation, particularly in the lateral and dorsal lobes, representing an alternative method. We also highlight the strengths and limitations of TP and sulpiride in replicating clinical symptoms and examine the toxicological effects of sulpiride on the kidney, testis, liver, and brain. We recommend the sulpiride model for the induction and studying of BPH, as it is readily accessible and closely mimics the pathogenesis of BPH in humans, unlike the TP model, which requires castration.

## 1. Introduction

Benign prostatic hyperplasia (BPH) is a common condition in older men, characterised by progressive enlargement of both glandular and stromal tissues within the prostate, resulting in increased organ volume [1,2]. The prevalence of BPH continues to rise, particularly in low- and middle-income countries. Estimates regarding its global impact differ; however, recent analyses suggest that approximately 210 million males worldwide may be affected, with a projected 45% risk of developing BPH over the next 30 years and an annual incidence of 379,000 men aged over 55 [3,4]. BPH pathophysiology involves both increases in prostatic volume (static component) and smooth muscle tone (dynamic component) [5]. Hyperplasia primarily occurs in the region surrounding the proximal urethra, where glandular expansion leads to bladder outlet obstruction (BOO). This obstruction frequently results in lower urinary tract symptoms (LUTSs), significantly diminishing patient quality of life. Common LUTSs include increased urinary frequency, urgency, and nocturia. As BPH progresses, more serious urinary complications may arise, including urinary retention, bladder diverticula, hydronephrosis, bladder stones, and renal insufficiency [1,6].

**Figure 1:**
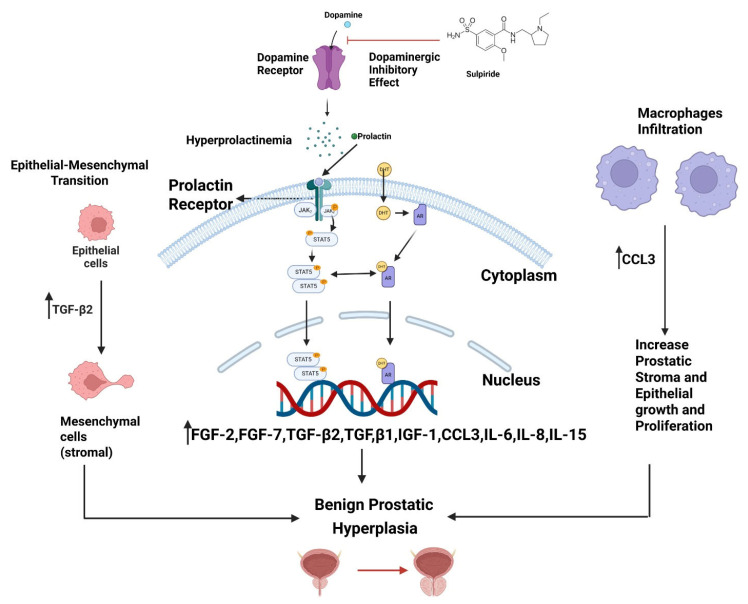
Mechanistic overview of sulpiride-induced benign prostatic hyperplasia (BPH) through the hypothalamic–pituitary and prostatic signalling pathway. Sulpiride blocks dopamine receptors, preventing dopamine’s inhibitory effect on prolactin release and causing hyperprolactinemia, which then activates prolactin receptors in prostatic cells, triggering JAK2–STAT5 signalling to promote nuclear transcription and prostatic epithelial and stromal proliferation. An increased prolactin level further stimulates stromal–epithelial interactions, inducing mesenchymal cells to release growth and inflammatory mediators, such as FGF-2, FGF-7, TGF-β1, TGF-β2, IGF-I, CCL3, IL-6, IL-8, and IL-15, which enhance epithelial–mesenchymal transition, stromal expansion, and macrophage infiltration. Together with androgen-dependent DHT–AR activation, these pathways synergistically increase prostatic epithelial and stromal growth, contributing to the development of BPH. Created in BioRender. Abdullah Sanusi. (2025). https://BioRender.com/ezywthp.

Sulpiride, known chemically as 2-methoxy-N-((1-propylpyrrolidine-2-yl)methyl)-5-sulphamoyl-benzamide, is a selective blocker of central dopamine receptors (D2, D3, and D4) [7]. It is mainly prescribed for schizophrenia, depression, and certain gastrointestinal disorders. Sulpiride exists as two optical enantiomers: (+) sulpiride and (−) sulpiride. Of these, the levorotatory (−) form, L-sulpiride, is more pharmacologically active within the central nervous system [8]. Side effects from doses at or above 600 mg/day include insomnia, fatigue, rapid heartbeat, liver problems, and tardive dyskinesia. By antagonising dopamine receptors, sulpiride also increases serum prolactin (PRL) levels [9], as shown in Figure 1, which outlines the role of hypothalamic–pituitary interactions and prostate signalling in how sulpiride induces BPH in animal models. PRL contributes to the growth and development of BPH [9], making sulpiride a common agent for inducing BPH in animal research.

Conversely, testosterone propionate (TP) is commonly employed to induce benign prostatic hyperplasia (BPH) in castrated rodent models. The standard protocol involves administering daily subcutaneous TP injections for approximately 4 weeks. This restoration of androgen (TP) stimulation elicits a robust proliferative response in the prostate, particularly within the ventral lobes, which demonstrate the most significant morphological changes. Histological examination of the prostate of experimental animals injected with TP consistently reveals features characteristic of BPH, including epithelial thickening, enlargement of glandular structures, and pronounced epithelial hyperplasia—closely paralleling the pathology observed in humans [10,11,12]. This review examines the use of TP and sulpiride to induce BPH, highlighting the strengths and limitations of each agent in replicating clinical symptoms. The sulpiride-induced model is considered advantageous due to its accessibility, ease of administration, and its ability to reproducibly induce BPH-like histopathological changes in experimental animals [9].

In contrast, TP’s controlled status as a Schedule III drug in the United States and a Class C drug in the UK presents challenges for experimental use. In addition, the sulpiride model of BPH is non-invasive (does not require castration) and therefore closely mimics the pathogenesis of BPH in humans, unlike the TP model, which involves castration of the experimental animal. Throughout this review, we survey the literature on both sulpiride and TP to compare their effectiveness in rodent models of BPH.

## 2. Methodology

This narrative review integrates evidence from basic science research that a single systematic review could not encompass. The search included studies from January 1977 to July 2025, covering both foundational and recent mechanistic research. Literature was retrieved from PubMed/MEDLINE, Embase, and Web of Science using combinations of MeSH and free-text terms, such as (“Benign prostatic hyperplasia induction” OR “rodent models”) AND (“Testosterone Propionate model”) AND (“sulpiride model”). Studies were selected if they used testosterone or sulpiride models to induce BPH. Studies were excluded if they were case reports that did not provide mechanistic or epidemiological information, were not in English without available translations, or had sources that could not be retrieved or verified. Titles and abstracts were screened for relevance, with eligible articles undergoing full-text review and thematic data extraction. No formal risk-of-bias scoring was performed; instead, studies were assessed by design, rigor, plausibility, and quality of evidence. As only published and publicly available data were reviewed, ethical approval was not required. Data were extracted based on key parameters—BPH induction, testosterone propionate, sulpiride, and rodent models—using thematic analysis. We compared findings across relevant studies to determine consensus, discrepancies, and evidence gaps. Instead of formal risk-of-bias tools, we assessed studies on design, sample size, methodological quality, biological plausibility, and alignment with existing evidence due to this review’s narrative approach. A thematic narrative approach was employed to synthesise the evidence, integrating epidemiological trends with mechanistic insights to develop a cohesive model for alternative methods of inducing and investigating BPH in rodent models. As this study relied exclusively on previously published data and publicly accessible reports, ethical approval was not necessary.

## 3. Androgens and Prolactin in BPH

Hormonal regulation is the main factor behind prostate growth in BPH. Research involving both humans and animal models has shown that androgens—especially testosterone and dihydrotestosterone (DHT)—are key drivers of prostate enlargement. At the same time, prolactin (PRL) may further encourage prostate cell growth. Nicholson and Ricke [13] reviewed evidence highlighting that signalling through the androgen receptor (AR) is essential for BPH. They pointed out that significant data connect androgens and AR activity to both prostate growth and the persistence of BPH; this connection is supported by the effectiveness of 5α-reductase inhibitors, which block the conversion of testosterone to DHT. Clinical evidence showing these drugs reduce prostate size and improve symptoms further strengthens this view [14]. The effectiveness of these medications underscores the critical role of ongoing androgen-AR signalling in BPH. Androgens promote the differentiation and proliferation of both stromal and epithelial cells in the prostate, and changes in steroid balance—such as shifts in the ratio between androgens and estrogens—can alter interactions between stromal and epithelial cells and lead to hyperplasia.

La Vignera et al. noted that age-related hormonal changes, such as declining testosterone levels or an altered androgen-to-estrogen ratio, contribute to a tissue micro-environment conducive to BPH onset [15]. Building on this, Tong and Zhou [16] demonstrated a link between androgens and inflammatory signalling in BPH, reporting that dihydrotestosterone (DHT) found in BPH tissue may activate chronic inflammatory responses in the prostate. This process amplifies pro-inflammatory cytokine expression and enhances the proliferative capacity of prostate cells. These findings indicate that a localised increase in DHT level can induce inflammation, which subsequently promotes hyperplasia. Such evidence supports a model wherein androgen-driven growth, and inflammatory pathways act collaboratively in the development of BPH [17,18]. Using the probastin-prolactin (Pb-PRL) transgenic mouse model, these researchers investigated the interaction between prolactin and androgen receptor (AR) in BPH. Genetic ablation of AR in prostate stromal (fibromuscular) cells markedly reduced PRL-induced prostatic hyperplasia, with both stromal and epithelial cell proliferation diminished in AR-deficient prostates compared to wild-type controls. Mechanistically, the study demonstrated that prolactin (PRL) mediates epithelial–stromal crosstalk by acting through epithelial PRL receptors to upregulate granulocyte–macrophage colony-stimulating factor (GM-CSF), which subsequently stimulates stromal cell proliferation via STAT3 signalling. Their findings established that prolactin-driven prostate growth depends on the presence of the androgen receptor (AR) in the stroma and identified a PRL/PRLR/GM-CSF/stromal proliferation axis in benign prostatic hyperplasia (BPH).

Dos Santos et al. [19] in their recent investigation utilising the Pb-PRL transgenic mouse model, revealed that this model recapitulates numerous histologic features characteristic of human BPH. Single-cell analysis indicated that PRL-induced BPH involves the expansion of epithelial cell subpopulations with diminished androgen signalling, likely originating from the reprogramming of normal luminal cells. Functionally, prostates from Pb-PRL mice exhibited increased vulnerability to oxidative stress. Treatment of these mice with an antioxidant led to reduced prostate weight and improvement in urinary symptoms, as well as inhibition of proliferation in PRL-driven epithelial organoids. These results suggest that PRL-driven hyperplasia can activate non-AR–dependent pathways—such as stress response and stemness programs in BPH, thereby implicating additional mechanisms beyond classic androgen action [19]. Taken together, these studies demonstrate that both androgens and prolactin (PRL) influence prostate cell growth. Androgens—especially DHT—bind to androgen receptors (AR), stimulating the proliferation of both stromal and epithelial cells; this underlies the use of 5α-reductase inhibitors in the treatment of BPH.

Additionally, DHT may create a local inflammatory environment that further encourages cell growth. Prolactin, via the prolactin receptor (PRLR), can activate pathways that promote growth and expand stromal tissue, as evidenced in PRL transgenic mouse models. Notably, the androgen and PRL systems interact: AR signalling increases PRL and PRLR expression in prostate cells, and higher blood PRL is associated with elevated AR levels. This bidirectional feedback means that, in BPH, androgens may make the prostate more responsive to prolactin’s effects—and vice versa.

## 4. Hyperprolactinemia

Hyperprolactinemia is characterised by an increase in the serum prolactin level beyond the normal range [20]. It is caused by several effectors, including the inhibitory action of antipsychotics such as sulpiride on the dopaminergic type 2 receptor on the surface of lactotrophs [21], specialised cells responsible for prolactin secretion, leading to drug-induced prolactin secretion. Hyperprolactinemia has been linked to various human health conditions, notably galactorrhea, ovarian dysfunction, vaginal mucosa, and neuroendocrine disorder [22]. The side effects of hyperprolactinemia are more pronounced in females than in males, postulated to be caused by the estrogen-mediated increase in prolactin [23]. In females, hyperprolactinemia is implicated in diminished gonadal function [8], enlargement of the breast, ovarian dysfunction, and has been hypothesised to be associated with the development of mammary duct ectasia [22]. In male subjects, hyperprolactinemia has been linked to erectile dysfunction, reduction in semen volume, and reduced libido [24]. To reduce the risk of hyperprolactinemia and potential adverse side-effects, especially in schizophrenia patients on sulpiride therapy, a dose decrease of the anti-psychotic is highly recommended [22], or alternative anti-psychotic drugs with less risk of hyperprolactinemia, such as Aripiprazole, Lurasidone, Quetiapine, or Asenapine should be dispensed [23].

The role of hyperprolactinemia in the development of benign prostatic hyperplasia (BPH) has been extensively studied [25,26,27,28]. Sulpiride-induced, hyperprolactinemia-driven BPH is a well-established model for studying BPH and investigating potential therapeutic modalities [19,29,30] due to the known age-related increase in prolactin in men and laboratory animals. Other findings from sulpiride-induced hyperprolactinemia studies have demonstrated a dose-dependent increase in height, weight, and the number of epithelial cells in the prostate [9,28]. While the exact mechanism of BPH emergence is unclear [31] in TP- and/or sulpiride-induced BPH models, the role of prolactin and its receptors in the prostate gland acinar epithelium in the activation of numerous signal pathways involving STAT3 and STAT5 transcription factors in regulating cell transition in the mitotic cycle has been reported in the development of prostate epithelium hyperplasia [32].

## 5. Testosterone Propionate (TP)-Induced BPH Model

In laboratory research, TP is commonly used to induce BPH in rodents, modelling the androgen-dependent aspect of human BPH [33], and male Sprague–Dawley or Wistar rats are a typical model for BPH induction. To remove endogenous androgens, rats are often bilaterally castrated before hormone treatment [12]. Following recovery, the castrated rats receive daily TP (an injectable testosterone ester) by subcutaneous injection. A standard protocol is TP 3–5 mg/kg body weight per day for 28 days to induce BPH [33]. Several studies have administered low doses of TP (3–5 mg/kg) to experimental animals over extended periods ranging from five to seven months, while higher doses (10–25 mg/kg) have typically been utilised for shorter durations, such as fourteen days [34,35,36]. At the end of TP treatment, rats are sacrificed, and the prostate, especially the ventral lobe, is dissected. Prostate weight, absolute and relative to body weight, is recorded, and tissues are fixed for histology/immunostaining compared with the vehicle-treated control groups. This protocol reliably produces prostate enlargement. TP causes marked prostate growth relative to intact or sham-operated rats. It is considered a well-established model of BPH in rodents [37].

## 6. Mechanism of TP-Mediated BPH Induction

**Figure 2:**
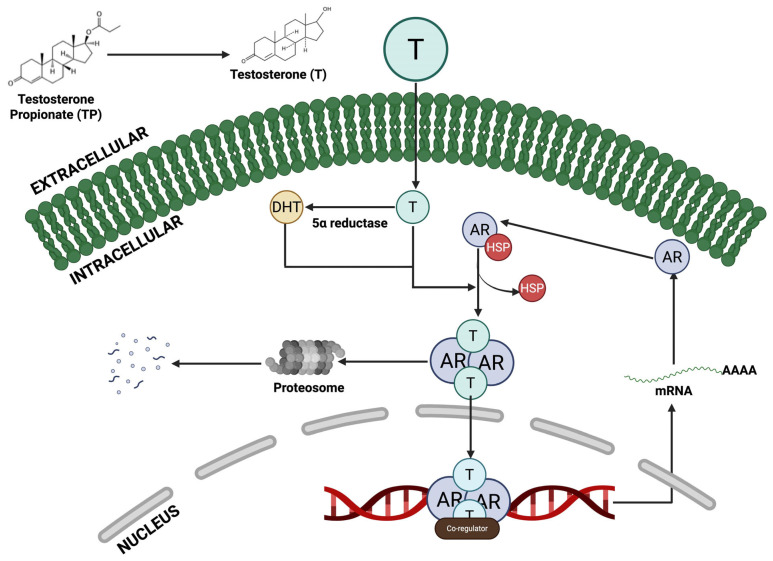
Mechanism of Testosterone Propionate/Testosterone-Induced AR Signalling in Prostate Cells. Testosterone propionate (TP) converts to testosterone (T), which then diffuses across the plasma membrane into the cytoplasm. Inside the cell, T can be metabolised into dihydrotestosterone (DHT) by 5α-reductase, a more potent androgen. Both T and DHT bind to the androgen receptor (AR), which is initially sequestered in the cytoplasm by heat shock proteins (HSPs). Ligand binding causes AR to change shape, HSPs to dissociate, and AR to dimerise, allowing it to move into the nucleus. In the nucleus, AR dimers bind to androgen response elements (AREs) on DNA and recruit co-regulators to activate transcription of androgen-responsive genes, resulting in mRNA production and protein synthesis. Disruption of this pathway plays a role in the development of benign prostatic hyperplasia (BPH) and the progression of prostate cancer (PCa). Created in BioRender. Uche O. Arunsi (2026). https://BioRender.com/.

TP itself is a testosterone ester; once injected, it is rapidly converted to testosterone, which is then reduced to dihydrotestosterone (DHT) by 5α-reductase in the prostate [38]. DHT is the most potent androgen in the gland. Both testosterone and DHT bind to the AR in the prostate epithelial and stromal cells. Upon androgen binding (Figure 2), AR translocate to the nucleus and drives gene expression of growth factors and proliferative signals [39,40]. In effect, the TP regimen massively boosts local androgen/AR signalling, mimicking the hormonal driver of human BPH [41]. The resulting AR activation stimulates the proliferation of prostate cells. The TP-induced BPH results in epithelial hyperplasia (marked expansion of the glandular epithelium) and stromal hyperplasia. Studies note that aberrant androgen/AR signalling potently drives the stromal compartment (which makes up 85–90% of the gland) and thereby promotes overall prostate enlargement [42]. In summary, exogenous TP increases circulating testosterone, which is converted to DHT; the DHT–AR complex then acts as a transcription factor to accelerate prostate cell proliferation.

## 7. Advantages and Limitations of the TP-Induced BPH Model

The TP model specifically reproduces the androgenic component of prostatic enlargement. It reliably yields prostate overgrowth and epithelial hyperplasia like those seen in men under excess androgen stimulation [33]. Because it is simple and reproducible, it is widely used to test anti-androgen or anti-proliferative therapies [12]. However, by design, this model focuses only on hormonal effects. It employs supraphysiologic androgen doses and (often) surgically removed testis, which do not fully mimic natural ageing BPH. It ignores non-androgenic factors, such as chronic inflammation, metabolic changes, and growth factor dysregulation, that also contribute to human BPH. The androgen model is not suitable for nonhormonal therapy studies and employs high hormone doses that do not accurately mimic the actual disease state. Moreover, rat prostates differ anatomically from those of humans (multiple lobes, varying stromal/epithelial ratios), and rats do not typically develop lower urinary tract symptoms [33]. Thus, TP-induced BPH is best viewed as a model of androgen-dependent hyperplasia rather than a complete recapitulation of clinical BPH.

## 8. Sulpiride-Induced BPH Model

Experimental sulpiride-induced BPH is generated in intact male rats (no castration), typically late-reproductive-age animals, and often Wistar strain [28]. Induced chronic hyperprolactinemia by treating sham-operated Wistar rats with sulpiride (40 mg/kg/day). Other protocols have given sulpiride orally or i.p. at 40–120 mg/kg daily for 4–6 weeks. Zheng et al. treated Brown-Norway rats orally with 40 or 120 mg/kg/day for 4 weeks and saw marked prostate enlargement [9]. Borovskaya et al. injected Wistar rats with 40 mg/kg subcutaneously (or i.p.) for 60 days [43], and Lesovaya et al. used 30 mg/kg i.p. for 28 days [44]. Despite these high doses, the model remains robust; all sulpiride-treated rats develop prostatic hyperplasia in the lateral lobes (and sometimes the dorsal lobes) [45]. In practice, only intact male rats are used. No castration is performed because sulpiride acts by augmenting the animal’s hormonal milieu. Sulpiride-mediated D2 blockade raises prolactin levels above normal androgen levels, which are sufficient to drive BPH [28].

## 9. Mechanism of Action

Sulpiride is a dopamine D_2_ receptor antagonist. By blocking D_2_ receptors on pituitary lactotrophs, it disinhibits PRL secretion. Chronic sulpiride causes hyperprolactinemia, which in turn stimulates prostate growth [9,45]. Elevated prolactin exerts mitogenic and anti-apoptotic effects on prostate cells. Sulpiride-treated rats exhibit increased expression of proliferation markers (e.g., PCNA) and anti-apoptotic proteins (Bcl-2) in the prostate [9]. At the receptor level, prolactin upregulates sex-steroid receptors in the gland, including the AR and estrogen receptor α (ERα). After sulpiride treatment, both AR and ERα levels increase, while ERβ levels tend to decline. This shift amplifies hormonal stimulation of growth. For instance, increased AR levels may drive local production of growth factors (e.g., IGF-1 in the stroma) and epithelial proliferation. Van Coppenolle et al. demonstrated that hyperprolactinemia leads to significant Bcl-2 overexpression in the lateral prostate, thereby suppressing apoptosis [28]. Similarly, Transforming Growth Factor-β (TGF-β) signalling is activated, and sulpiride markedly increases TGF-β mRNA in the prostate [45], consistent with stromal expansion and extracellular matrix deposition. In summary, sulpiride increases prolactin, which then promotes both stromal and epithelial proliferation by boosting AR/ERα–driven mitogenic signalling and enhancing anti-apoptotic pathways (e.g., Bcl-2) and pro-growth factors (e.g., TGF-β) in the prostate [9].

## 10. Advantages and Limitations of Sulpiride-Induced BPH

A key advantage of the sulpiride model is that it recapitulates non-androgenic drivers of BPH. Because intact animals are used, the endocrine environment remains physiological; no exogenous hormones or castration are required. This makes the model especially relevant for studying BPH associated with hyperprolactinemia or other non-DHT stimuli. Indeed, sulpiride-induced BPH mimics drug-induced hyperprolactinemia in humans (e.g., psychiatric patients on D_2_ blockers) and the age-associated rise in prolactin [32].

Importantly, unlike classic testosterone/DHT models, this system does not rely on 5α-reductase activation. For example, standard therapy with finasteride (a DHT blocker) does not prevent sulpiride-driven growth or inflammation; by contrast, rapamycin (which targets cell proliferation pathways) is effective [44]. In other words, the sulpiride model is “androgen-independent”, and prostate enlargement occurs despite normal DHT levels. This is also a limitation; pathways dependent on DHT (and thus many BPH therapies) are not engaged in this model. Hence, one cannot test 5α-reductase inhibitors or castration effects here. Other limitations include the artificial nature of high-dose sulpiride, which may present off-target effects and interspecies differences [44].

Nevertheless, by using non-castrated rats, the sulpiride model preserves the natural hormone interplay and has proven to be reproducible across many studies. It provides a valuable complement to androgen-driven models, highlighting the role of prolactin and stromal signalling in BPH [28,33].

## 11. Comparisons of TP- and Sulpiride-Induced BPH Models

In rat models of benign prostatic hyperplasia (BPH), testosterone propionate (TP) and sulpiride induce prostatic growth via distinct hormonal routes. Critically, the experimental setup differs: TP-induced BPH is typically generated in castrated rats, whereas sulpiride-induced BPH is generally performed in intact (non-castrated) animals. For example, Li et al. (2018) [38] castrated male rats and then injected TP (25 mg/kg subcutaneously) once daily for four weeks to induce BPH. Similarly, Lee et al. used castrated Sprague–Dawley rats with TP (3 mg/kg/day, s.c.) for 28 days to model BPH. In contrast, sulpiride models do not require castration [12]. Indeed, Tahiri-Zagret and Oria found that sulpiride prostatic effects were blunted or absent in castrated rats; intact males showed marked gland enlargement, but castrated males showed almost no change. Thus, castration is required for the TP model (to eliminate endogenous androgen and then reintroduce a defined androgen stimulus). In contrast, the sulpiride model relies on hyperprolactinemia acting within an intact androgen milieu [46].

The primary hormones involved differ as well. Androgens drive TP models; exogenous testosterone propionate (TP) raises systemic testosterone and its potent metabolite, dihydrotestosterone (DHT). DHT, generated by 5α-reductase in the prostate, strongly activates the androgen receptor (AR). Jung et al. demonstrated that TP-treatment (with no additional estrogen) enlarged rat prostates and elevated both DHT levels and AR signalling. By contrast, the sulpiride model is driven by prolactin (PRL). Sulpiride chronically elevates pituitary PRL levels [47]. Zheng et al. confirmed that 4-week sulpiride treatment significantly increased serum PRL (along with FSH and even testosterone), and this hyperprolactinemia produced prostate enlargement. In essence, the TP model exemplifies testosterone/DHT-driven hyperplasia. In contrast, the sulpiride model exemplifies a prolactin-mediated hyperplasia (with prolactin acting as a paracrine prostatic growth factor on top of background androgens) [9].

Despite different upstream signals, both models converge on androgen receptor (AR) signalling in the prostate. In the TP model, AR is directly activated by high androgen levels. DHT-AR binding induces proliferation and survival gene programs. For instance, Jung et al. noted that TP-implanted rats had upregulated AR expression and androgen-responsive co-activators in prostate tissue. In the sulpiride model, AR is engaged more indirectly. Prolactin can potentiate AR signalling. Zheng et al. found that sulpiride-treated rats showed AR upregulation in the lateral lobe [9]. Moreover, Van Coppenolle et al. showed that sulpiride-induced prostate growth required androgen support. In castrated rats, prolactin delivered via sulpiride alone did not induce growth; a growth response occurred only when testosterone (T) or DHT was additionally supplied [28,48]. This indicates that PRL-driven hyperplasia is androgen-dependent. Mechanistically, both models increase prostatic survival signals. TP-BPH is associated with a higher Bcl-2/Bax ratio, while Van Coppenolle also reported that chronic hyperprolactinemia induced Bcl-2 overexpression in the lateral prostate [28]. In summary, AR involvement is crucial in both models, but TP acts as the direct AR ligand, whereas sulpiride-induced PRL amplifies AR signalling (and possibly ER signalling as well).

Estrogen signalling via ERα also diverges: in the TP-derived estradiol model, estradiol predominantly engages ERα through local aromatisation, whereas in the sulpiride-induced hyperprolactinemia model, PRL may indirectly enhance ERα activation by upregulating aromatase or ER expression, thereby modifying the pattern and magnitude of estrogen-dependent responses [49]. Interestingly, Jung et al. reported that TP treatment alone increased ERα expression in hyperplastic prostate tissue (along with AR) [47]. However, this ERα upregulation is secondary to excess androgen and local aromatisation. By contrast, the sulpiride model strongly induces ERα. Zheng et al. observed that the lateral prostates of sulpiride-treated rats showed marked ERα overexpression (and ERβ downregulation) [9]. Thus, sulpiride-BPH features a hormonal milieu that is relatively high in estrogenic signalling (perhaps due to prolactin effects on aromatase and estrogen receptor regulation). In short, TP-BPH is mainly androgenic (with only modest ERα induction), whereas PRL-driven BPH notably upregulates ERα.

Both models are instructive for different aspects of human disease. The TP model shows the centrality of androgens; clinical BPH is AR-dependent (5α-reductase inhibitors shrink human BPH). The sulpiride (hyperprolactinemia) model highlights additional modulators. Clinical data show that prolactin receptors are present in the human prostate, and PRL can act as a prostatic mitogen. Prostate-specific PRL overexpression in transgenic mice causes BPH-like growth. Thus, while TP-BPH recapitulates pure androgenic growth, sulpiride-BPH models a scenario in which hormonal imbalance (high PRL in the setting of androgens) drives mixed stromal-epithelial enlargement. These mechanistic insights (AR vs. PRL/ERα pathways, epithelial vs. stromal proliferation) help in translating animal studies to human BPH pathophysiology and therapy.

## 12. Toxic Effects of Sulpiride on the Testis

Abd, Al-Juhaishi, and Jumma [50] conducted a controlled, in vivo study to examine the impact of sulpiride—a dopamine D_2_ receptor antagonist on testicular structure and reproductive function in adult male rats. The study employed 30 Wistar male rats, aged 90–95 days and weighing 250–300 g, which were randomly assigned to three equal groups (*n* = 10 per group). Group 1 received 10 mg/kg of sulpiride, Group 2 received 25 mg/kg, and Group 3 served as the control (receiving normal saline), with all treatments administered via gavage over a defined period. Within 24 h after treatment, blood samples were collected under isoflurane anaesthesia to measure serum levels of testosterone, prolactin, luteinising hormone (LH), and follicle-stimulating hormone (FSH). Concurrently, testes and epididymides were harvested; left testes underwent histopathological analysis, while right testes plus epididymides were frozen to assess sperm parameters. Hormonal outcomes revealed dose-dependent endocrine disruption. Specifically, rats in the 25 mg/kg group (Group 2) exhibited a significant decrease in testosterone and LH (*p* < 0.05), as well as a significant increase in prolactin; FSH levels remained unchanged across all groups. Sperm analysis demonstrated that Group 2 exhibited significantly reduced sperm concentration and motility, along with increased sperm abnormalities and higher percentages of dead sperm, compared to both the lower-dose group and controls (*p* < 0.05). Histological examination further confirmed a dose-dependent deterioration of seminiferous tubule architecture. Moderate degeneration was observed in Group 1 (10 mg/kg). At the same time, severe tubule destruction was evident in Group 2, characterised by loss of spermatogenic cells and structural disruption, in contrast to the intact morphology seen in control rats. The study’s outcomes suggest that sulpiride induces hyperprolactinemia via D_2_ receptor antagonism, which, in turn, suppresses the hypothalamic–pituitary–gonadal axis. The reduction in GnRH (Gonadotropin-Releasing Hormone) release likely caused the decline in LH and testosterone, culminating in hypogonadism, impaired spermatogenesis, and testicular damage. Through comprehensive hormonal assays, sperm metrics, and histopathological evaluations, this study provides robust evidence that even moderate levels of sulpiride can produce significant testicular and reproductive harm in adult male rats [50].

Vieira et al. [51] conducted a pivotal experimental study to assess the long-term effects of maternal lactational exposure to sulpiride on the reproductive and behavioural development of male rat offspring. The study examined whether sulpiride, a dopamine D_2_ receptor antagonist that may cause hyperprolactinemia and pass into breast milk, affects suckling pups. Lactating Wistar rats were split into three groups: control, 2.5 mg/kg/day sulpiride, and 25 mg/kg/day sulpiride, all treated orally from postnatal day 1 to 21. These doses simulated low and high clinical exposures. Male offspring were monitored until PND 90 to assess long-term reproductive and behavioural effects. At PND 90, histological and morphometric analysis showed dose-dependent testicular damage, with reduced seminiferous tubule volume, germinal epithelium height, and Leydig cell count in both sulpiride-exposed groups. Notably, the 25 mg/kg group exhibited a higher frequency of abnormal seminiferous tubules, suggesting substantial disruption to spermatogenesis and testicular architecture. This indicates that early-life exposure may lead to long-term, potentially irreversible changes. Vieira et al. concluded that lactational exposure to sulpiride causes long-term damage to male reproductive organs, even in the absence of behavioural changes. The results underscore the risks of using prolactin-elevating antipsychotics during lactation and highlight the testis as a critical target organ during developmental drug exposure [51].

Clomiphene citrate (CC) test—measures the hypothalamic drive to increase endogenous GnRH and subsequent LH release.

Luteinising hormone-releasing hormone (LHRH) test—evaluates pituitary capacity to secrete LH in response to GnRH.

Human chorionic gonadotropin (hCG) test—assesses testicular Leydig cell responsiveness in testosterone production.

The CC test results indicated a progressive decline in LH response: while the ratio of LH released post-CC at 2 weeks of sulpiride treatment remained near normal (0.942 ± 0.073), by 60 days it had significantly decreased to 0.769 ± 0.121 (*p* < 0.05 vs. 2 weeks; *p* < 0.01 vs. baseline). This finding suggests that prolonged hyperprolactinemia inhibits hypothalamic GnRH release, thereby diminishing LH secretion over time. While LHRH and hCG test data were mentioned, the primary conclusion centred on the CC test, with less emphasis on pituitary and testicular responses. Nonetheless, impairment of LH release at the hypothalamic level implies that downstream effects, including reduced LH stimulation of Leydig cells, are likely to occur. The study, therefore, highlights the hypothalamus as an initial site of action where chronic prolactin elevation begins to impair HPT axis function, even if gonadal function remains promptly intact. Oseko et al. concluded that in healthy men, chronic sulpiride-induced hyperprolactinemia progressively suppresses the hypothalamic component of the HPT axis, primarily via reduced GnRH release. This effect shows dysfunction at the pituitary or testicular level [52].

The cumulative evidence from animal and human studies confirms that sulpiride, a dopamine D_2_ receptor antagonist, exerts profound, dose-dependent adverse effects on male reproductive health, primarily by disrupting the hypothalamic–pituitary–gonadal (HPG) axis. Abd, Al-Juhaishi, and Jumma [50] demonstrated that sulpiride administration in adult male rats led to a cascade of hormonal and structural testicular impairments. The dose-dependent hyperprolactinemia induced by sulpiride corresponded with a significant suppression of luteinizing hormone (LH) and testosterone levels, along with clear histological evidence of seminiferous tubule degeneration and sperm abnormalities. These findings strongly implicate prolactin-mediated suppression of gonadotropin-releasing hormone (GnRH) as a central mechanism leading to hypogonadism, impaired spermatogenesis, and testicular damage. Complementary insights from Vieira et al. [51] underscore the long-term reproductive consequences of early-life sulpiride exposure. Maternal sulpiride administration during lactation caused irreversible testicular damage in male offspring by adulthood, as evidenced by reduced seminiferous tubule volume and disrupted germinal epithelium architecture. This suggests that developmental exposure during critical windows of sexual maturation poses heightened risks, even when behavioural parameters remain unchanged. The findings emphasise that prolactin-elevating antipsychotics can affect reproductive tissue permanently, with testicular structure and spermatogenesis being especially vulnerable. Human data from Oseko et al. [52] further affirm this mechanistic pathway, showing that chronic sulpiride use in healthy men results in progressive suppression of LH release due to impaired hypothalamic GnRH activity. While pituitary and testicular functions initially appeared to be preserved, the decline in LH responsiveness following clomiphene stimulation provides early evidence of HPG axis dysregulation. This finding is critical because it validates animal models and confirms that hyperprolactinemia can be a silent but progressive threat to male fertility, even in clinical settings. Taken together, these studies establish a coherent model: sulpiride-induced hyperprolactinemia disrupts reproductive function by suppressing hypothalamic GnRH output, thereby impairing pituitary LH secretion and downstream testosterone synthesis. This hormonal imbalance culminates in histological testicular degeneration and spermatogenic failure. The reproductive risks associated with sulpiride are therefore both dose and duration-dependent, and the timing of exposure, especially during developmental windows, amplifies long-term harm. These insights highlight the need for clinical vigilance when prescribing prolactin-elevating antipsychotics to reproductive-age men and lactating women, and advocate for regular hormonal monitoring to pre-empt irreversible testicular dysfunction.

## 13. Toxic Effects of Testosterone Propionate on the Testis

The induction of glandular overgrowth in experimental models of Benign Prostatic Hyperplasia (BPH) is conventionally done by the use of exogenous testosterone propionate (TP) [53,54]. However, the resultant supraphysiological levels of androgen have a paradoxical effect of toxicity on the testes. In comparison to dopamine antagonists, such as sulpiride that impair testicular functioning through prolactin-mediated suppression [50], TP-induced toxicity is mediated by direct endocrine feedback and localized oxidative insult [55].

The chronic administration of TP initiates a potent negative feedback loop on the hypothalamic–pituitary–gonadal (HPG) axis. TP suppresses the secretion of luteinizing hormone (LH) and follicle-stimulating hormone (FSH) from anterior pituitary by imitating elevated levels of systemic androgens [56,57]. This causes a functional castration condition as the Leydig cell-mediated endogenous testosterone production is halted, often leading to an extensive testicular atrophy and decreased organ-to-body weight ratios [58].

A severe toxic effect of TP-induced BPH induction is the alteration in the Blood–Testis Barrier. Exogenous androgens in high levels have the potential to disrupt tight junctions between the Sertoli cells. This elevated permeability permits the inflammatory indicators and reactive oxygen species (ROS), which compromises the specialized microenvironment necessary in the maturation of germ cells [59]. TP-induced toxicity is strongly mediated by an imbalance in the testicular redox status. With the length of exposure, malondialdehyde (MDA) accumulation and the corresponding reduction in catalase and superoxide dismutase (SOD) activity occur [60]. This oxidative condition triggers the Bax/Bcl-2 apoptotic intrinsic mechanism in the seminiferous tubules, causing the destruction of spermatogonia and a significant decrease in the sperm concentration and sperm viability [61].

TP-induced testicular toxicity is histologically distinguished by the shrinkage of seminiferous tubules and loss of the interstitial space [62]. Studies have shown that there is usually a decrease in the height of the germinal epithelium and presence of vacuoles, which indicates a breakdown of cellular integrity [62,63]. These alterations differ with the hyperplasia observed in the prostate, highlighting the tissue-specific toxicodynamics of testosterone propionate.

## 14. Toxic Effects of Sulpiride on the Liver

### 14.1. Evidence of Hepatotoxicity

#### Clinical Case Studies

A 2023 case report in the Scholars Journal of Medical Case Reports described a 24-year-old male who developed fatigue and jaundice after four weeks of sulpiride (100 mg/day) for anxiety. Liver enzymes were markedly elevated (ALT: 550 IU/L, AST: 490 IU/L), accompanied by increased bilirubin levels. Investigations for viral, autoimmune, and metabolic liver diseases were negative. Imaging was normal, and liver biopsy showed eosinophilic and lymphocytic infiltration, consistent with immuno-allergic drug-induced liver injury (DILI). Management involved discontinuation of sulpiride and weekly monitoring, resulting in full recovery within 1 month. This case highlights the need for vigilance even with drugs of low hepatotoxic potential [64].

Research, particularly in animal models, suggests that sulpiride may contribute to metabolic abnormalities that, in turn, indirectly affect liver health. Studies in rats have shown that chronic administration of sulpiride can induce fatty liver disease by phosphorylating insulin receptor substrate-1 (IRS-1) at Serine 307, resulting in insulin resistance in adipose tissue. This mechanism highlights a potential link between sulpiride, insulin resistance, and hepatic triglyceride accumulation, which could contribute to broader liver dysfunction [65].

A 59-year-old woman developed jaundice and signs of liver damage after ten months on sulpiride. A liver biopsy revealed cholestatic hepatitis and ductopenia, a loss of bile ducts, suggesting a destructive cholangitis. The absence of typical allergic reactions, such as rash or fever, pointed to a toxic rather than an immune-mediated drug effect, possibly due to a genetic predisposition affecting sulpiride metabolism. Despite discontinuing sulpiride and starting supportive treatment with cholestyramine, vitamin K, and vitamin D, her jaundice persisted, leading to evaluation for a liver transplant [66].

## 15. Management

Early detection and immediate withdrawal of the offending drug remain the cornerstone of managing sulpiride-induced liver injury. Since there is no specific antidote for most cases of drug-induced liver injury (DILI), treatment typically focuses on supportive care. This may involve relieving symptoms such as jaundice and itching and, in more severe presentations, addressing complications such as impaired blood clotting or hepatic encephalopathy. In rare but serious scenarios, such as progressive liver failure or persistent cholestasis that evolves into biliary cirrhosis, liver transplantation may become the only life-saving option, as seen in the reported case of the 59-year-old woman who developed bile ductopenia after long-term sulpiride therapy.

## 16. Toxic Effects of Sulpiride on the Kidney

### 16.1. Renal Elimination and Risk of Accumulation

The primary route of excretion of sulpiride is the kidney, and it is excreted unchanged [67,68]. Multiple pharmacokinetic studies have confirmed that the hepatic metabolism of sulpiride is negligible, and the drug is primarily eliminated through glomerular filtration and active tubular secretion [69]. Consequently, any impairment in renal function, whether due to age-related decline or underlying disease, may result in drug accumulation. In a risk assessment study of the effect of sulpiride on patients with schizophrenia, it was observed that the elderly group, despite administration of lower doses, had a lower clearance of sulpiride compared to the younger group. This was attributed to a physiological decline in glomerular filtration rate [8].

### 16.2. Experimental and Clinical Evidence of Renal Effects

The renal effects of sulpiride have been studied in both animal models and human volunteers. One of the functions of dopamine is to promote the excretion of sodium in the urine to regulate blood pressure. However, sulpiride antagonises dopamine, thus inhibiting its role in sodium balance. A study examined the dissociation between sulpiride effects on renal hemodynamics and its influence on natriuresis during dopamine infusion. In this study, sulpiride significantly reduced urinary sodium excretion (FENa+). Still, it did not significantly alter baseline renal plasma flow (ERPF), glomerular filtration rate (GFR), or filtration fraction (FF). The effect of sulpiride on natriuresis suggests interference with dopaminergic regulation of tubular function, particularly proximal tubular sodium handling [70]. These findings were consistent across individuals pretreated with alpha-adrenergic blockers (prazosin or phentolamine), suggesting that sulpiride’s natriuretic antagonism was independent of alpha-adrenergic mechanisms. Instead, the effect was attributed to sulpiride’s antagonism of dopamine at renal tubular DA1 and DA2 receptors, which mediate vasodilation, natriuresis, and inhibition of aldosterone secretion [70].

Interestingly, while sulpiride did not interfere with dopamine-induced vasodilation, its suppression of sodium excretion was robust, indicating a more pronounced effect on dopamine’s tubular actions than on its vascular effects. The anti-natriuretic effect occurred without significant changes in renal perfusion parameters, suggesting a localised tubular action. This reinforces the hypothesis that sulpiride can affect electrolyte handling by antagonising endogenous dopamine, even at therapeutic concentrations [70].

In a study comparing the effects of sulpiride and clozapine (another antipsychotic drug) on kidney function, renal function tests showed that sulpiride alone had no significant effect on serum creatinine, blood urea nitrogen, urea, or uric acid levels, unlike clozapine. It was observed that sulpiride alone induced mild congestion of the peritubular capillaries with cloudy swelling of the epithelial lining of some renal tubules, accompanied by obliteration of the lumen. Sulpiride in combination with clozapine showed mild histological lesions as evidenced by mild vacuolation of the epithelial lining of the renal tubules. Co-administration attenuated clozapine-induced nephrotoxicity, suggesting a modulatory interaction, albeit without direct sulpiride toxicity, which may indicate that effects vary when sulpiride is used in combination or may instead confer protection under certain conditions [68].

In a study to check for the response of an isolated rat kidney to a mixed solution of amino acids using sulpiride as one of the three renal autocoid inhibitors, sulpiride was found to partially inhibit the effects of the mixed amino acid solution on inulin clearance and renal perfusate flow and that it entirely inhibited the reduction in fractional albumin excretion [71].

### 16.3. Interaction with Dopaminergic Pathways

Sulpiride interference with renal dopaminergic pathways is increasingly recognised as central to its potential renal effects. Dopamine, produced locally in the kidney, plays a vital role in sodium excretion, blood pressure regulation, and overall renal homeostasis. Sulpiride, by antagonising dopamine receptors, may disrupt this balance, particularly under conditions requiring compensatory natriuresis (e.g., volume overload, high salt intake).

Furthermore, studies in animal models have suggested that sulpiride may exhibit dose-dependent antagonism of dopamine-induced renal vasodilation, although human data remain inconclusive. Variation in sulpiride impact on the renal vasculature across species and study designs underscores the need for caution when extrapolating preclinical findings to clinical settings. In a study investigating the effects of dopamine and sulpiride on regional blood flow in the rat kidney, sulpiride was found to reduce renal cortical blood flow [72]. A study in humans demonstrated that (+) sulpiride is a dopamine receptor antagonist in the kidney, resulting in reduced renal blood flow, increased sodium excretion, and increased glomerular filtration, thereby interfering with dopamine activity [73]. Agnoli and colleagues demonstrated that sulpiride, particularly the L-enantiomer, blocks dopamine’s beneficial renal effects (vasodilation, increased GFR, natriuresis) by antagonising D_2_ receptors [74].

### 16.4. Pharmacokinetics and Risk of Accumulation

Unlike many antipsychotics, nearly all sulpiride is excreted unchanged via the kidneys. In early pharmacokinetic studies, approximately 70% of a dose was recovered unmodified in human urine. With renal impairment, the elimination half-life and systemic exposure increased substantially, while renal clearance diminished, necessitating dose reductions of 35–70% or extended dosing intervals in such patients. This places elderly patients or those with chronic kidney disease at significant risk of drug accumulation and potential toxicity [67]. In a study to understand the distribution of sulpiride in the body and its pharmacodynamics using Positron Emission Tomography (PET), it was observed that the kidneys showed lower sulpiride uptake than other organs, such as the bladder, liver, and gallbladder. Sulpiride was also found not to be properly cleared by the kidneys in mice with organic cation transporters [75].

### 16.5. Role of Renal Transporters

A breakthrough study demonstrated that sulpiride is a substrate for and is managed by human carnitine OCT2, organic cation transporters 1 and 2 (OCTN1/2), and human multidrug and toxin extrusion protein 1 and 2 (hMATE1/2-K), transporters integral to proximal tubular secretion. OCT2 mediates the uptake of sulpiride from the bloodstream to the proximal tubule cells/renal cells. At the same time, MATEs facilitate the efflux of sulpiride from proximal tubular cells into the renal lumen, allowing for excretion into urine, and OCTN participates in both renal excretion and absorption. Disruption or saturation of these systems, due to drug interactions or genetic polymorphisms, may increase intrarenal accumulation and systemic retention. The accumulation of sulpiride was reduced in mouse primary renal tubular cells by OCT2 and OCTN. This study suggests that polypharmacy scenarios involving OCT2/MATE inhibitors (e.g., cimetidine) could further impede sulpiride elimination [75,76].

### 16.6. Cellular Pathways and Molecular Effects

Recent mechanistic work reveals that sulpiride disrupts canonical pathways in proximal tubular cells. In a study investigating the interaction between the dopamine D_2_ receptor (D2R) and the Wnt/β-catenin signalling pathways, Sulpiride was found to decrease β-catenin phosphorylation and enhance TCF/LEF transcriptional activity, potentially altering cellular proliferation and repair responses [77]. This suggests that chronic D_2_ blockade may perturb renal cellular homeostasis, increasing susceptibility to injury or maladaptive repair.

### 16.7. Case Reports and Clinical Implications

A case report revealed that Sulpiride, when taken in higher doses, may trigger a cascade of reactions that severely affect the kidneys. After the withdrawal of Sulpiride, the renal function returned to normal [78]. A reported case described a 46-year-old man who developed neuroleptic malignant syndrome, characterised by hyperpyrexia, severe muscle rigidity, altered consciousness, autonomic instability, rhabdomyolysis, and focal tubulitis on renal biopsy, culminating in acute myoglobinuric renal failure following the discontinuation of sulpiride and maprotiline [79]. In a documented case of sulpiride overdose, a 23-year-old male with schizophrenia ingested an excessive dose of sulpiride. He subsequently developed severe rhabdomyolysis and acute renal failure, requiring hospitalisation [80].

## 17. Toxic Effects of Testosterone Propionate on the Kidney

When administered in supraphysiological dosages, testosterone propionate (TP) may have major off-target effects on the kidney [81,82]. According to some studies, increased androgen exposure may be linked to renal structural alterations, fibrosis, tubular damage, and functional impairment, despite the fact that androgens can have complicated, context-dependent effects in renal tissues [82,83]. Two ways via which androgens can negatively impact the kidney are renal fibrosis and extracellular matrix buildup [82]. Chronic TP administration (2 mg/kg/day for 84 days) was linked to profibrotic signaling via the transforming growth factor-β1 (TGF-β1)/Smad pathway, increased expression of extracellular matrix proteins (such as collagen I and collagen IV), and thickening of the glomerular basement membrane in aged male rats [84]. These results show that TP can affect important pathways linked to renal fibrosis and altered kidney architecture, and that TP treatment attenuates age-related fibrosis by decreasing TGF-β1/Smad signaling while activating antioxidant Nrf2-ARE pathways [84].

Additionally, in vitro analysis showing androgen-induced apoptosis of renal tubular epithelial cells suggested that the HIF-1α/BNIP3 pathway is activated as a cause of cell death [83,85]. This implies that testosterone, then TP, may cause tubular damage and heightened vulnerability to tubular cell death under specific pathophysiological circumstances [85]. Experimental models show that androgens can affect kidney functional and hemodynamic characteristics. Studies in sodium-loaded rats show that androgen exposure through testosterone supplementation is linked to increased blood pressure, decreased creatinine clearance, and increased microalbuminuria [86]. These findings are consistent with functional impairments in renal handling of solutes and glomerular filtration [86]. This evidence suggests that androgen excess plays a part in kidney stress and injury, even if it is not specific to TP [81,82,86].

In susceptible mice, androgen-mediated renal consequences may involve oxidative stress, inflammation, and activation of profibrotic signaling networks outside traditional androgen receptor pathways, all of which contribute to chronic kidney injury [81,82]. Also, research indicates that sex hormone therapy, which includes the administration of testosterone, may affect human renal blood flow, inflammatory biomarkers, and structural integrity, and the results rely on the underlying illness context and hormone levels [87].

## 18. Toxic Effect of Sulpiride on the Brain

Schizophrenia is a complex neuropsychiatric syndrome that comprises a group of severe mental illnesses of unknown aetiology. The basis for the pathology of schizophrenia is the hyperfunction of the dopamine (DA) system in the mesolimbic and mesocortical pathways, accompanied by metabolic disorders of serotonin (5-hydroxytryptamine, 5-HT) [88]. Somatoform disorders are a group of conditions that comprise physical symptoms for which no adequate medical explanation can be found. These somatic complaints are severe enough to cause persistent distress for the patient and to impair their ability to function in everyday life. They are also likely to be associated with the development of other mental disorders. The diagnostic class of somatoform disorders is relatively new, being first introduced into the Diagnostic and Statistical Manual of Mental Disorders (DSM) classification in 1980 [89].

### 18.1. Mechanism of Action and Limitation

Dopamine is the primary catecholamine neurotransmitter in the brain, with its receptors located on both presynaptic and postsynaptic membranes. Sulpiride is believed to primarily modulate the Mesocorticolimbic dopamine system (MCLDA), which serves as the primary reward pathway [90]. Sulpiride administration can produce complex effects, influenced in part by the anatomical location of dopamine D2 receptors (D2R), whether presynaptic or postsynaptic [91]. Lower doses of sulpiride primarily block presynaptic D2/D3 auto receptors, leading to increased dopamine release and synthesis in some brain areas [90,92]. In contrast, higher doses are thought to mainly inhibit postsynaptic D2 receptors, thereby reducing dopamine release and signalling within the brain [93]. Consequently, elevated dosages of sulpiride may be employed to manage schizophrenia, a condition associated with excessive dopaminergic activity. Mohyeldin et al. [94] report that sulpiride exerts its antidepressant effects primarily by selectively inhibiting dopamine and serotonin receptors across the brain. Compared to other antidepressant agents, sulpiride has attracted considerable attention due to its favourable safety profile, minimal extrapyramidal side effects, reduced affinity for non-target neuronal receptors, demonstrated efficacy, and cost-effectiveness.

### 18.2. Clinical Cases

Peng et al. [95] conducted a study utilising sulpiride to examine its effects on reversing chronic corticosterone (CORT) exposure in mice. Chronic CORT administration resulted in anxiety-like behaviours and impaired food-seeking, attributed to reduced excitability and diminished excitatory synaptic transmission in ventral tegmental area (VTA) dopamine neurons. This reduction was associated with elevated somatodendritic dopamine levels that activated inhibitory D2 receptor signalling. Direct administration of sulpiride into the VTA restored neuronal excitability and glutamatergic transmission onto dopamine neurons. In behavioural assessments, sulpiride alleviated anxiety-like symptoms and rescued CORT-induced deficits in food-seeking behaviour within mildly aversive environments, although it did not improve operant motivation task performance. These results indicate that sulpiride mitigates the inhibitory action of excess dopamine via D2 receptors, offering potential therapeutic strategies for stress-related neuropsychiatric conditions. A study by Rouillon et al. [89] examining the effects of sulpiride on somatoform disorders found that sulpiride predominantly inhibits limbic dopamine receptors, exhibiting minimal impact on striatal dopamine receptors. This selective receptor activity likely contributes to its low incidence of extrapyramidal side effects. At lower doses, sulpiride preferentially antagonises presynaptic receptors, thereby increasing dopaminergic transmission, which may underlie its activating properties and broad therapeutic efficacy. Its effectiveness in treating somatoform and functional disorders is attributed to its capacity to selectively block dopamine activity in regions such as the area postrema, the hypothalamic-hypophyseal system, and the mesolimbic pathways [89]. Depicted in Table 1 is the summary of the toxic effect of sulpiride on the organs mentioned above as indicated by previously published studies.

## 19. Toxic Effect of Testosterone Propionate on the Brain

Induction of benign prostatic hyperplasia (BPH) through chronic administration of testosterone propionate (TP) in rodents for experimental research, elevates systemic testosterone and dihydrotestosterone (DHT) levels [96]. While the prostate is the primary target tissue, TP-induced BPH models inevitably engage the central nervous system (CNS) due to the tight regulation of androgen signaling by the hypothalamic–pituitary–gonadal (HPG) axis [97]. In rodents, exogenous TP disrupts normal endocrine feedback by suppressing hypothalamic gonadotropin-releasing hormone (GnRH) and pituitary luteinizing hormone (LH) secretion [98]. This neuroendocrine suppression alters androgen receptor (AR) expression and signaling within brain regions involved in hormonal homeostasis, including the hypothalamus, amygdala, and hippocampus [99,100]. Such changes are particularly relevant in BPH models, where prolonged androgen exposure is sustained to drive prostate enlargement.

Testosterone and its metabolite DHT readily cross the blood–brain barrier, allowing TP to exert direct central effects in BPH-induced rodents [101]. Androgen receptors expressed in neurons and glial cells regulate transcriptional programs linked to neuroplasticity, neurotransmitter balance, and oxidative stress responses. Studies using TP-induced BPH models have shown increased systemic oxidative stress and inflammation, which may secondarily affect the brain by promoting neuroinflammatory signaling and altering redox balance within neural tissue [102]. Furthermore, chronic androgen exposure in rodent BPH models has been associated with modulation of dopaminergic and serotonergic pathways, particularly within limbic and reward-related brain regions [103]. Although these effects are not always the primary endpoints of BPH studies, evidence from TP-treated rodents suggests alterations in dopamine turnover and serotonin signaling, which may influence behavior, motivation, and stress responsiveness. These neurochemical changes are best understood as secondary consequences of prolonged androgen excess, rather than direct neurotoxicity. Importantly, TP-induced BPH models also demonstrate changes in estrogenic signaling in the brain, as testosterone undergoes aromatization to estradiol [99]. This shift in androgen–estrogen balance may further influence hypothalamic regulation and neuroendocrine control, and reinforce prostate growth while simultaneously affecting CNS function.

## 20. Limitations and Gaps in the Reviewed Studies

Beyond hormones, immune and inflammatory pathways are poorly characterised in BPH models. Chronic inflammation is a hallmark of human BPH [104] but most TP- or sulpiride-based studies do not quantify immune mediators. Several reports indicate pronounced inflammation when these models are challenged. For example, Bello et al. [105] found that a high-salt diet in TP-treated Wistar rats markedly elevated pro-inflammatory cytokines (IL-6, IL-8) and COX-2 expression in the prostate [105]. Similarly, Van Coppenolle et al. [28] demonstrated that chronic sulpiride administration (40 mg/kg) induces lateral lobe enlargement accompanied by inflammation in Wistar rats. Zhang et al. [104] showed in an autoimmune prostatitis (EAP) model that inflammation drives stromal expansion. EAP-treated rats exhibited significantly more CD3^+^ T-cell and CD68^+^ macrophage infiltration, higher TGF-β_1_ and RhoA/ROCK signalling, and increased extracellular matrix deposition compared to hormone-only rats [104]. These findings imply that even androgen-induced BPH models should be profiled for immune activity.

Future studies should therefore systematically measure inflammatory cytokines (e.g., IL-1β, IL-6, TNF-α, IL-17), chemokines, and cyclooxygenases; leukocyte infiltrates (T cells, macrophages, mast cells) by IHC or flow cytometry; and oxidative stress markers (ROS levels, MDA, antioxidant enzymes). For instance, Zhang et al. [88] noted that TP treatment alone elevates oxidative/hypoxic stress (increased HIF-1α, ROS), which can, in turn, trigger inflammation. Profiling both TP- and sulpiride-induced prostates in parallel would clarify how each model engages the immune axis. In summary, a significant gap exists in the lack of integrated immunophenotyping in BPH models, which often lack cytokine panels and immune cell counts, thereby missing opportunities to connect rat models to the inflamed microenvironment of human BPH.

## 21. Conclusions

The testosterone propionate (TP) and sulpiride models induce BPH in rats via distinct mechanisms. TP drives prostatic overgrowth via classic androgen signalling: exogenous testosterone is locally converted to dihydrotestosterone, activating the androgen receptor and yielding marked epithelial hyperplasia in the ventral prostate lobe. By contrast, sulpiride causes hyperprolactinemia, which preferentially stimulates proliferation in the lateral (and dorsal) lobes. In the sulpiride model, both epithelial and stromal compartments expand—for example, stromal markers such as vimentin and fibronectin are strongly upregulated in lateral lobes—and inflammatory changes (immune-cell infiltration) are evident in the lateral prostate. Thus, TP-induced BPH is predominantly an androgen-driven, ventral-epithelial hyperplasia, whereas sulpiride-induced BPH is a prolactin-driven proliferation of both stroma and epithelium in the lateral lobes.

The choice of model should be tailored to the research question. For studies targeting androgenic mechanisms, such as testing 5α-reductase inhibitors or anti-androgen compounds, the TP model is most appropriate, as it elicits pronounced AR-mediated epithelial growth. In contrast, investigations of prolactin signalling, estrogenic crosstalk, or inflammation-related pathways will benefit from the sulpiride model. This model induces prolactin-dependent lateral-lobe hyperplasia and an inflammatory microenvironment, neither of which occurs in the TP paradigm. By aligning the model with the biological axis of interest, investigators can more directly translate findings to human BPH. In summary, TP- and sulpiride-induced BPH in rats are distinct yet complementary.

## Figures and Tables

**Table 1:** Summary of Research Findings on Sulpiride Toxicities in the Testis, Liver, Kidney, and Brain.

S/N	Study/Model	Findings	References
1	In vivo controlled study in adult male Wistar rats	High doses of sulpiride (25 mg/kg) increased prolactin, reduced testosterone and LH, caused low sperm count, poor motility, abnormal forms, and severe testicular damage, while low-dose effects were milder and controls remained normal.	[50]
2	Maternal sulpiride and testis damage in male offspring	With sulpiride (25 mg/kg), male offspring from sulpiride-treated mothers showed dose-dependent testis damage, with smaller seminiferous tubules, thinner germ cell layers, and fewer Leydig cells.	[51]
3	Controlled clinical study in healthy adult males	Sulpiride (300 mg/day for 78 days) caused sustained hyperprolactinemia (71.6–95.3 ng/mL) in 4/5 participants.	[52]
4	An animal study (rat model)	Chronic administration of sulpiride induced fatty liver disease by increasing phosphorylation of insulin receptor substrate-1 (IRS-1) at Serine 307, leading to insulin resistance in adipose tissue, promoting hepatic triglyceride accumulation.	[65]
5	A case study	Sulpiride (100 mg/day) increased ALT, AST, and bilirubin levels, along with eosinophilic and lymphocytic infiltration.	[64]
6	A case study	A patient developed jaundice and severe liver injury after 10 months on sulpiride. Liver biopsy revealed cholestatic hepatitis with ductopenia, indicating destructive cholangitis.	[66]
7	A case study	Sulpiride causes a marked reduction in sodium excretion by blocking renal DA1 and DA2 receptors, impairing dopamine-mediated natriuresis without affecting renal blood flow or filtration.	[70]
8	Animal study comparing sulpiride vs. clozapine	Sulpiride alone did not cause significant changes in kidney function markers but did produce mild renal tubular injury, characterised by peritubular congestion and cloudy swelling of tubular cells. When combined with clozapine, it caused only mild tubular vacuolation and even appeared to lessen clozapine-related nephrotoxicity.	[68]
9	An isolated rat kidney study	Sulpiride completely inhibited the amino acid–induced reduction in fractional albumin excretion.	[71]
10	An animal study	Sulpiride reduced renal cortical blood flow.	[72]
11	A case study	Sulpiride reduced renal blood flow, increased sodium excretion, and GFR.	[73]
12	A case study	L-Sulpiride blocked dopamine-induced vasodilation, GFR increase, and natriuresis.	[74]
13	A case study	A 46-year-old man developed neuroleptic malignant syndrome after stopping sulpiride and maprotiline, showing hyperpyrexia, muscle rigidity, altered consciousness, autonomic instability, and acute myoglobinuric renal failure indicated by focal tubulitis on renal biopsy.	[79]
14	A case study	Severe rhabdomyolysis and acute renal failure following sulpiride overdose in a 23-year-old male with schizophrenia, requiring hospitalisation.	[80]
15	An animal study	Sulpiride restored neuronal function, reduced anxiety-like behaviour, and rescued food-seeking, but not operant motivation.	[95]
16	A Clinical Study	Sulpiride shows selective limbic dopamine blockade with minimal striatal effect, explaining low EPS risk. Low doses block presynaptic receptors, enhancing dopaminergic transmission.	[89]

## Data Availability

No new data were created or analyzed in this study. Data sharing is not applicable to this article.
